# Spirituelle Kompetenz in Psychiatrie und Psychotherapie – Hindernisse und Erfolgsfaktoren

**DOI:** 10.1007/s00115-020-00975-0

**Published:** 2020-08-10

**Authors:** Eckhard Frick, Philip Ziemer, Stephan Heres, Karl Ableidinger, Franz Pfitzer, Arndt Büssing

**Affiliations:** 1 Klinik und Poliklinik für Psychosomatische Medizin und Psychotherapie, Klinikum rechts der Isar, Technische Universität München, Ismaninger Str. 22, 81675 München, Deutschland; 2 kbo-Isar-Amper-Klinikum gGmbH, Akademisches Lehrkrankenhaus der LMU München, Vockestraße 72, 85540 Haar bei München, Deutschland; 3 Landesklinikum Amstetten-Mauer, Hausmeninger Str. 221, 3362 Mauer, Österreich; 4 Klinik Chiemseewinkel, Römerstraße 17, Seeon-Seebruck, 83358 Deutschland; 5 Professur für Lebensqualität, Spiritualität und Coping, Universität Witten/Herdecke, Alfred-Herrhausen-Str. 50, Witten, 58448 Deutschland

**Keywords:** Kompetenz, Spiritual Care, Psychiatrie, Psychotherapie, Implementierung, Spiritual care, Competency, Psychiatry, Psychotherapy, Implementation

## Abstract

**Hintergrund:**

Ebenso wie die World Psychiatric Association (WPA) und andere nationale psychiatrische Fachgesellschaften hat auch die Deutsche Gesellschaft für Psychiatrie und Psychotherapie, Psychosomatik und Nervenheilkunde (DGPPN) ein Positionspapier zu Religiosität und Spiritualität (R/Sp) in Psychiatrie und Psychotherapie veröffentlicht, in dem sie Patientenzentrierung und spirituelle Kompetenz (SpK) der psychiatrischen Berufe fordert. Es ist bekannt, dass Kompetenzmangel das wichtigste Hindernis für die Implementierung von Spiritual Care (SpC) in die klinische Praxis darstellt.

**Fragestellung:**

Ziel der vorliegenden Studie ist die praxisnahe Untersuchung der SpK in Psychiatrie und Psychotherapie. Wie schätzen psychiatrisch Tätige die SpK ihrer eigenen Berufsgruppe ein und welche Variablen beeinflussen dieses Urteil?

**Material und Methoden:**

Insgesamt 391 psychiatrische Pflegekräfte, 75 Psychiater, 119 Therapeuten verschiedener Professionen und 62 andere (*n *= 647) aus Krankenhäusern in Deutschland und Österreich füllten den SpC Competency Questionnaire (SCCQ) aus.

**Ergebnisse:**

Pflegekräfte, ältere und spirituell erfahrenere Personen schätzen die SpK der eigenen Berufsgruppe vergleichsweise höher ein und meinen seltener, diesbezüglich nicht zuständig zu sein. Pflegende nennen häufiger als andere Berufsgruppen das Fehlen geeigneter Räumlichkeiten als Hindernis für die Implementierung von SpC. Höhere Einschätzung der SpK der eigenen Berufsgruppe geht mit höheren Werten in den SCCQ-Faktoren „Selbsterfahrung und proaktive Öffnung“, „Team-Spirit“, „Wahrnehmungs‑/Dokumentationskompetenz“ einher.

**Diskussion:**

Die Zuständigkeit der Gesundheitsberufe für SpC in Psychiatrie und Psychotherapie wird unter den deutschsprachigen psychiatrischen Berufsgruppen noch kontrovers diskutiert. Dies hängt mit mangelnder Kompetenz in diesem Feld zusammen.

## Abstract (en)

**Background:**

Just as the World Psychiatric Association (WPA) and other national psychiatric societies, the German Association for Psychiatry, Psychotherapy and Psychosomatics (DGPPN) has published a position statement about religiosity and spirituality in psychiatry and psychotherapy, in which it demands patient orientation and spiritual competency in psychiatric professions. Previous research has shown that lack of competency is the major barrier against implementing spiritual care into clinical practice.

**Objective:**

The aim of this study was to examine spiritual care in psychiatry and psychotherapy. An evaluation of how health professionals in psychiatry gauge the spiritual care competency of their professional group and which variables influence this judgement.

**Material and methods:**

A total of 391 psychiatric nursing personnel, 75 psychiatrists, 119 therapists from diverse professions and 62 others, i.e. 647 working in German and Austrian hospitals completed the German version of the spiritual care competency questionnaire (SCCQ).

**Results:**

Nursing personnel, older and spiritually more experienced persons gauged the spiritual competency of their own professional group comparatively higher and judged less frequently that they have no responsibility in this field. Nursing personnel reported the lack of suitable rooms as a barrier against implementation of spiritual care more often than other professional groups. Judging the spiritual competency of one’s own professional group higher is associated with higher values in the SCCQ factors self-experience and proactive opening up, team spirit, perception and documentation competency.

**Conclusion:**

The responsibility of healthcare professions for spiritual care in psychiatry and psychotherapy is still a controversial issue among German-speaking psychiatric professional groups. This is partially due to a lack of competency in this domain.

## Hintergrund

Das DGPPN-Positionspapier zum Umgang mit Religiosität (*R*) und Spiritualität (*Sp*) in Psychiatrie und Psychotherapie [29] formuliert Anforderungen und Grenzen hinsichtlich der Einbeziehung religiös(*r*)-spiritueller(*sp*) Elemente in die Behandlung. Mangelnde Kenntnisse und fehlende spirituelle Kompetenz (*SpK*) werden immer wieder als Hindernisse genannt, die der Implementierung von Spiritual Care (*SpC*) im Wege stehen [1–3, 20, 30]. Die Positionspapiere der DGPPN [29] und der WPA [18] machen deutlich, dass in der aktuellen psychiatrischen Fort- und Weiterbildung die Wissensvermittlung bezüglich *R/S* und deren Auswirkungen auf das Erleben psychisch kranker Menschen zu wenig berücksichtigt werden.

Curlin und Kollegen [8] stellten bei US-amerikanischen Psychiatern (Rücklaufquote 63 %) fest, dass sich diese im Vergleich zu anderen medizinischen Fachgruppen seltener als *r* bezeichnen, sie schilderten aber in der Behandlung von Patienten sowohl häufiger Spontanberichte als auch die eigene proaktive Exploration dieses Erlebnisbereiches. Eine Befragung aller erreichbaren niedergelassenen Psychotherapeuten im nordbayerischen Raum (*n* = 1081, Rücklaufquote 65 %; [17]) zeigte, dass ca. zwei Drittel der Therapeuten *R* für ein wichtiges Thema in der Psychotherapie halten, aber nur ca. ein Fünftel routinemäßig eine *sp* Anamnese erhebt. Eine solche *sp* Kurzanamnese ist insbesondere im US-amerikanischen Raum in vielen Bereichen etabliert [24] und wird zunehmend auch im deutschsprachigen Raum praktiziert [12]. Die eigene Nähe zur *R* korreliert positiv mit der Tendenz, die Kurzanamnese in die Therapie einzubinden. Bei ca. einem Drittel der Therapeuten besitzt *R* einen wichtigen Stellenwert innerhalb der eigenen Weltanschauung („personal bias“; [17]).

Freund und Kollegen [11] befragten psychiatrische Weiterbildungsermächtigte in Deutschland. Insgesamt bezeichneten sich 60 % der antwortenden Weiterbildungsermächtigten selbst als *r* und/oder *sp*. Der Großteil der Befragten misst der Integration von *sp* Fragestellungen in die Facharztweiterbildung eine hohe Bedeutung bei. Eine Signalwirkung kommt der Richtlinie des österreichischen Gesundheitsministeriums zu [15], die eindringlich vor der Gefahr des Missbrauchs *sp* Methoden in der Psychotherapie warnt.

Ein aktueller Handbuchbeitrag [21] beklagt den psychiatrischen Nachholbedarf im Vergleich zu *SpC* innerhalb der Palliativmedizin; es existiere noch immer kein Konsens über psychiatrische *SpC*-Kernkompetenzen. Stattdessen werde versucht, *r/sp* Patientenbelange an die Seelsorge zu delegieren.

Die aktuelle Studie erfragt erstmals im deutschen Sprachraum sowohl einzelne Bereiche der selbsteingeschätzten Spiritual-Care-Kompetenz (*SpCK*) aller psychiatrisch-psychotherapeutischen Berufsgruppen als auch das globale Urteil dieser Experten bezüglich der *SpK* ihrer Berufsgruppe. Mittels eines standardisierten und validierten Befragungsinstrumentes werden die folgenden Forschungsfragen bearbeitet:

1. Wie schätzen Psychiater und andere an der psychiatrischen Akutversorgung beteiligte Berufsgruppen ihre Zuständigkeit bzw. Nichtzuständigkeit für *SpC* ein?
2. Von welchen Variablen wird die Einschätzung fehlender Zuständigkeiten für *SpC* beeinflusst?
3. Wie unterscheiden sich die Berufsgruppen hinsichtlich der Gründe, die der Umsetzung von *SpC* im Wege stehen?

## Studiendesign und Untersuchungsmethoden

### Probanden und Rekrutierung

#### Rekrutierung

Psychiatrisch-psychotherapeutisch Tätige im Isar-Amper-Klinikum München (Rücklaufquote ca. 31 %), im Landesklinikum Mauer (39 %) und in drei Kliniken des Trägers Gesundheitswelt Chiemgau (60 %) wurden persönlich kontaktiert. Interessierte erhielten den Fragebogen in Papierform und wurden in der Folge einmal erinnert. Alle Teilnehmenden erklärten ihre Bereitschaft zur anonymisierten Verwendung der Daten.

#### Soziodemographische und berufsbezogene Daten

Alle Probanden wurden nach ihrem Geschlecht, Alter, Familienstand, Religionszugehörigkeit sowie nach Merkmalen ihrer beruflichen Tätigkeit gefragt. Außerdem konnten sie sich äußern, ob sie sich als aktiv gläubige Person bezeichnen würden (4-stufige Zustimmungsskala: ja, unbedingt; ja, etwas; eher nein; nein, gar nicht) oder wie häufig sie beten oder meditieren (regelmäßig; hin und wieder; eher selten; gar nicht).

### *SpC*-Kompetenzen

Zur Beurteilung der selbsteingeschätzten *SpCK* wurde der Spiritual Care Competence Questionnaire (*SCCQ*) verwendet [13]. Dieser nutzt 26 Items und differenziert 7 Dimensionen (Cronbachs α 0,73–0,86):

1. Wahrnehmungs*-K* (z. B. Bedürfnisse der Angehörigen oder Patienten wahrnehmen),
2. Team-Spirit (z. B. Austausch im Team über das Thema oder eigene *Sp*),
3. Dokumentations*-K* (z. B. Kenntnis von Instrumenten zur Erfassung *sp* Bedürfnisse, Fähigkeit zur nachvollziehbaren Dokumentation),
4. Selbsterfahrung und proaktive Öffnung (z. B. Vertiefung eigener *Sp*, Zuwendung zu Patienten, um deren *sp* Bedürfnisse anzusprechen),
5. Wissen über andere Religionen (z. B. Kenntnisse zu Besonderheiten verschiedener Religionsgemeinschaften und deren Berücksichtigung),
6. Gesprächsführungs*-K* (z. B. offenes Gespräch über existenzielle oder religiöse Themen führen können),
7. proaktive Empowerment*-K* (z. B. Ermöglichung der Teilnahme an *r* Feiern, geeigneter Rahmen für *sp* Gespräche).

Umsetzungshindernisse für *SpC* wurden mit 4 zusätzlichen Items erfasst (Cronbachs α 0,72: Ich weiß zu wenig über *R/Sp*, um mich kompetent einbringen zu können; keine Zeit für *r/sp* Themen; kein geeigneter Raum vorhanden, um geschützt über *r/sp* Themen zu sprechen; empfinde mich für *r/sp* Themen als nicht zuständig). Für die Zustimmung bzw. Ablehnung der entsprechenden Aussagen des *SCCQ* wurde eine 4‑stufige Skalierung gewählt (0 – stimmt nicht; 1 – stimmt kaum; 2 – stimmt eher; 3 – stimmt genau).

### Statistische Analysen

Die deskriptive, Varianz, Korrelations- (Spearman ρ) und stufenweise Regressionsanalysen wurde mit dem Programm SPSS in der Version 23.0 durchgeführt. Es wurde ein Signifikanzniveau von <0,01 gewählt. Für die Korrelationsanalysen wurde eine Korrelationskoeffizient *r* > 0,5 als starke Korrelation angesehen, *r* zwischen 0,3 und 0,5 als moderate Korrelation, *r* zwischen 0,2 und 0,3 als schwache Korrelation und *r* < 0,2 als keine oder vernachlässigbare Korrelation.

## Ergebnisse

### Beschreibung der teilnehmenden Personengruppe

Es konnten Datensätze von 647 Personen ausgewertet werden: 12 % Ärzte, 60 % Pflegende, 18 % andere (Therapeuten, Psychologen). Diese Berufsgruppen unterscheiden sich signifikant sowohl in Bezug auf Alter und Geschlecht, Arbeitszeit und Berufstätigkeit als auch bezüglich der Religionszugehörigkeit und der Häufigkeit *r* Praxis (Tab. 1). Im Schnitt haben 33 % keinerlei Religionszugehörigkeit, der Anteil der Katholiken ist bei den Ärzten deutlich geringer und der Anteil der Protestanten etwas höher; die Hälfte der untersuchten Personen bezeichnet sich als „gläubig“.

**Table 1:** 

	Alle	Ärzte	Pflegende	Andere	Signifikanz (ANOVA/χ^2^)
*Anzahl*^a^	647	75	391	119	–
*Alter (Jahre)*	41,5 ± 11,8	45,9 ± 10,5	40,9 ± 11,9	40,8 ± 11,6	0,003
*Frauen (%)*	73	73	69	84	0,007
*Berufstätigkeit (Jahre)*	19,0 ± 12,4	17,1 ± 10,4	20,3 ± 12,9	15,8 ± 11,5	0,001
*Arbeitszeit (Stunden/Woche)*	35,4 ± 9,2	39,7 ± 10,5	36,1 ± 8,3	30,4 ± 9,2	<0,0001
*Berufliche Zufriedenheit (1–5)*	4,0 ± 0,8	4,1 ± 0,7	3,9 ± 0,8	4,1 ± 0,7	0,023
*Religionszugehörigkeit (%)*					0,001
Katholisch	53	40	54	56	
Evangelisch	8	16	5	14	
Andere	7	8	6	7	
Keine	33	36	35	23	
*Gläubig (%)*	54	62	52	56	n. s.
*Häufigkeit Gebet/Meditation (1–4)*	2,3 ± 1,1	2,6 ± 1,1	2,2 ± 1,1	2,6 ± 1,1	<0,0001
**Kompetenzen (SCCQ)**					
Wahrnehmungs*-K*	1,9 ± 0,6	2,1 ± 0,6	1,9 ± 0,6	2,1 ± 0,6	<0,0001
Team-Spirit	0,8 ± 0,6	0,8 ± 0,6	0,8 ± 0,6	0,7 ± 0,5	n. s.
Dokumentations*-K*	0,6 ± 0,7	0,5 ± 0,6	0,7 ± 0,7	0,5 ± 0,6	0,002
Selbsterfahrung und Öffnung	1,0 ± 0,7	1,1 ± 0,7	0,9 ± 0,6	1,2 ± 0,7	<0,0001
Wissen über andere Religionen	1,7 ± 0,7	1,6 ± 0,6	1,7 ± 0,7	1,7 ± 0,6	n. s.
Gesprächsführungs*-K*	2,3 ± 0,7	2,5 ± 0,6	2,2 ± 0,7	2,4 ± 0,6	<0,0001
Empowerment*-K*	1,8 ± 0,6	1,9 ± 0,7	1,8 ± 0,6	1,9 ± 0,7	0,020
Umsetzungshindernisse	1,2 ± 0,8	1,0 ± 0,8	1,3 ± 0,7	1,0 ± 0,7	<0,0001

^a^62 Personen konnten keiner Profession zugeordnet werden

### Empfundene Zuständigkeit für *SpC*

In der Gesamtgruppe stimmen 27,5 % nicht zu, eine besondere *SpK* zu haben, 37,0 % stimmen kaum zu, 29,0 % stimmen eher zu und 6,5 % stimmen genau zu. Der verneinenden Aussage, dass ihre Berufsgruppe nicht für *SpC* zuständig sei, wurde in der Gesamtgruppe zumeist nicht zugestimmt: 37,0 % stimmten nicht zu, 27,7 % stimmen kaum zu, 22,3 % stimmen eher zu und 13,0 % stimmen genau zu.

Es zeigte sich ein Trend für besonders geringe Zustimmung bei den Ärzten, dass sie eine besondere *SpK* hätten (Tab. 2).

**Table 2:** 

		Meine Berufsgruppe hat besondere *SpK*	Meine Berufsgruppe ist nicht für *SpC* zuständig
Insgesamt	MW	1,15	1,08
	SD	0,87	1,04
	*n*	579	521
*Profession*			
Arzt	MW	0,91	0,93
	SD	0,86	0,95
	*n*	70	72
Pflege	MW	1,17	1,12
	SD	0,88	1,03
	*n*	373	324
Andere	MW	1,12	1,00
	SD	0,94	1,08
	*n*	114	103
F‑Wert		2,46	1,33
*p*-Wert		0,087	n. s.
*Altersgruppen*			
<31 Jahre	MW	1,02	1,13
	SD	0,87	1,02
	*n*	141	120
31–40 Jahre	MW	1,07	1,32
	SD	0,94	1,05
	*n*	140	131
41–50 Jahre	MW	1,23	1,03
	SD	0,84	1,07
	*n*	130	118
>50 Jahre	MW	1,27	0,89
	SD	0,91	0,98
	*n*	168	152
F‑Wert		2,76	4,39
*p*-Wert		0,041	0,005
*Gebet/Meditation*			
Nicht/selten	MW	1,08	1,27
	SD	0,89	1,03
	*n*	317	281
Hin und wieder/regelmäßig	MW	1,23	0,92
	SD	0,90	1,03
	*n*	282	254
F‑Wert		4,32	15,40
*p*-Wert		0,038	<0,0001

Dargestellt ist die Ausprägung der Variablen „besondere *SpK*“ bzw. „Nichtzuständigkeit“ in Bezug auf Profession, Alterskohorten und Häufigkeit der Gebets‑/Meditationspraxis

Für beide Aussagen gab es altersassoziierte Unterschiede: Mit zunehmendem Alter wurde eine besondere *SpK* gesehen bzw. wurde die Nichtzuständigkeit verneint (Tab. 2). Geschlechtsassoziierte Unterschiede ließen sich nicht finden (nicht dargestellt).

Personen, die häufiger beten oder meditieren, zeigen eine etwas höhere Zustimmung, eine besondere *SpK* zu haben als Personen, die dies nicht oder nur selten tun würden. Die Nichtzuständigkeit wird signifikant eher von den nicht bzw. wenig betenden oder meditierenden Personen benannt als von den „religiöseren“ Personen (Tab. 2).

### *SpC*-Kompetenzen der Berufsgruppen

Die selbsteingeschätzten *SpCK* zeigen deutliche Ausprägungsunterschiede (Tab. 1). Die Gesprächsführungs*-K* wird als hoch eingeschätzt (besonders von Ärzten, signifikant geringer von Pflegenden), ebenso die Wahrnehmungs*-K* (signifikant geringer bei Pflegenden) und Empowerment*-K* (mit geringen Unterschieden zwischen den Berufsgruppen). Deutlich geringe *K*-Zuschreibungen finden sich für Selbsterfahrung und Öffnung (signifikant höher bei den anderen Berufsgruppen und gering bei den Pflegenden), Team-Spirit (ohne signifikante Unterschiede zwischen den Berufsgruppen) und besonders gering für Dokumentations*-K* (besonders gering bei Ärzten und anderen Berufsgruppen).

Signifikante geschlechtsassoziierte Unterschiede lassen sich nicht finden (nicht dargestellt). Mit zunehmendem Alter steigen *K* im Bereich Selbsterfahrung und Öffnung (F = 13,5; *p* < 0,0001), Empowerment*-K* (F = 6,1; *p* < 0,0001) und das Wissen über andere Religionen (F = 4,0; *p* = 0,008) an.

Die Einschätzung als gläubiger bzw. wenig/nichtgläubiger Mensch ist mit unterschiedlich hoch ausgeprägten *SpCK* assoziiert: Selbsterfahrung und Öffnung (F = 109,9; *p* < 0,0001) und Wahrnehmungs*-K* (F = 13,3; *p* < 0,0001) sind bei Gläubigen deutlich stärker ausgeprägt, etwas weniger ausgeprägt auch Empowerment*-K* (F = 4,6; *p* = 0,033), Gesprächsführungs*-K* (F = 4,5; *p* = 0,034) und Dokumentations*-K* (F = 4,2; *p* = 0,041), während die Umsetzungshindernisse (F = 10,3; *p* = 0,001) bei den Wenig‑/Nichtgläubigen signifikant größer waren. Lediglich für Team-Spirit und Wissen über andere Religionen zeigen sich keine Gruppenunterschiede.

### Empfundene Zuständigkeit für *SpC* und Einflussvariablen

Wie hängt die empfundene Zuständigkeit bzw. Nichtzuständigkeit mit den *SpCK* und berufsbezogenen Variablen zusammen? In Korrelationsanalysen ergibt sich kein signifikanter Zusammenhang mit Berufsjahren, Arbeitszeit oder beruflicher Zufriedenheit (*r* < 0,12). Die selbsteingeschätzte „besondere *SpK*“ der Berufsgruppe ist moderat assoziiert mit Wahrnehmungs*-K*, Team-Spirit und Selbsterfahrung und Öffnung (*r* > 0,30, *p* < 0,0001); die Nichtzuständigkeit hat einen moderaten Zusammenhang mit Umsetzungshindernissen (*r* = 0,49; *p* < 0,0001) sowie moderat invers mit Selbsterfahrung und Öffnung (*r* = −0,412, *p* < 0,0001).

Die Bedeutsamkeit dieser assoziierten Variablen wird mithilfe einer Regressionsanalyse ausgewertet (Tab. 3). Für die besondere SpK sind 4 *SpCK* als Prädiktoren bedeutsam (21 % Varianzerklärung), wobei Selbsterfahrung/Öffnung alleine bereits 13 % der Varianz erklärt. Für die Nichtzuständigkeit sind Umsetzungshindernisse und Selbsterfahrung/Öffnung signifikante Prädiktoren, die 28 % der Varianz erklären (Umsetzungshindernisse bereits 24 %). In keinem der Regressionsmodelle waren die Alterskohorte oder die Häufigkeit von Gebet/Meditation von signifikanter Bedeutung.

**Table 3:** 

		R^2^	Beta	T	*p*
Abhängige Variable: Item 57 (besondere *SpK*)^a^ F = 37,3; *p* < 0,0001					
Modell 4	(Konstante)	–	–	1,319	0,188
	Selbsterfahrung und Öffnung	0,13	0,234	5,399	<0,0001
	Team-Spirit	0,18	0,169	4,090	<0,0001
	Dokumentations*-K*	0,20	0,121	2,916	0,004
	Wahrnehmungs*-K*	0,21	0,118	2,605	0,009
Abhängige Variable: Item 59 (Nichtzuständigkeit)^b^ F = 98,0; *p* < 0,0001					
Modell 2	(Konstante)	–	–	6,721	<0,0001
	Umsetzungshindernisse	0,24	0,375	8,675	<0,0001
	Selbsterfahrung und Öffnung	0,28	−0,235	−5,443	<0,0001

Dargestellt ist die hierarchische Bedeutsamkeit der eingeschlossenen Einflussvariablen auf die Ausprägung der abhängigen Variablen „besondere *SpK*“ bzw. „Nichtzuständigkeit“

^a^Nichtsignifikant im Modell: Alterskohorte, Häufigkeit Gebet/Meditation, Empowerment*-K*, Umsetzungshindernisse

^b^Nichtsignifikant im Modell: Alterskohorte, Häufigkeit Gebet/Meditation, Empowerment*-K*, Wahrnehmungs*-K*, Team-Spirit, Dokumentations*-K*

### Umsetzungshindernisse für *SpC*

Nach Hinderungsgründen gefragt, stimmen die meisten Befragten eher wenig zu (Tab. 4), was für Professionalität und Offenheit angesichts eines ungewohnten Themenbereiches spricht. Das Thema zu wechseln, wenn Patienten auf *r* Themen zu sprechen kommen, findet keinerlei Zustimmung. Mangelnde Zeit, um *sp* Themen mit Patienten anzusprechen, findet eine gewisse Zustimmung, ebenso der Wunsch, mehr Zeit zu haben, um mit Patienten über deren *sp* Bedürfnisse zu sprechen. Keine geeigneten Räumlichkeiten als Hinderungsgrund wurden signifikant eher von Pflegenden angegeben. Ein zu geringes Wissen oder keine Zuständigkeit für den Themenbereich hatte eher eine Bedeutung. Die fehlende Zuständigkeit wurde signifikant häufiger von den Pflegenden bejaht. Die abwehrende Aussage, nicht alles lösen zu können, spielt keine große Bedeutung im Kollektiv, fand aber signifikant eher bei den Ärzten Zustimmung.

**Table 4:** 

Scores 0–3		Unangenehm, über *sp* Themen zu reden	Wechsle Thema, wenn Patient auf *r* Themen zu sprechen kommt	Habe keine Zeit, *r/sp* Themen anzusprechen	Hätte gerne mehr Zeit, um mit Patienten über deren *sp* Bedürfnisse zu sprechen	Kein geeigneter Raum vorhanden	Weiß zu wenig	Nicht zuständig	Kann nicht alles lösen
Insgesamt (*n* = 584)	MW	0,65	0,01	1,17	1,31	1,16	1,20	1,21	0,22
	SD	0,85	0,13	1,05	0,96	1,10	0,96	1,03	0,74
Ärzte (*n* = 75)	MW	0,53	0,00	1,16	1,38	0,88	1,01	0,96	0,69
	SD	0,89	0,00	1,09	1,02	0,94	0,94	0,94	1,15
Pflegende (*n* = 381)	MW	0,68	0,01	1,21	1,29	1,29	1,27	1,32	0,06
	SD	0,86	0,14	1,04	0,97	1,12	0,95	1,04	0,40
Andere (*n* = 118)	MW	0,62	0,02	1,04	1,35	0,90	1,10	1,00	0,45
	SD	0,80	0,13	1,04	0,89	1,04	1,00	1,03	1,04
F‑Wert		1,04	0,39	1,16	0,40	*8,66*	2,92	*6,68*	*33,79*
*p*-Wert		n. s.	n. s.	n. s.	n. s.	*<0,0001*	0,055	*0,001*	*<0,0001*

Dargestellt ist der Einfluss der Profession auf die Ausprägung der Umsetzungshindernisse

## Diskussion

Die in der Einleitung formulierten Forschungsfragen können wir folgendermaßen beantworten:

1. Eine besondere Zuständigkeit für *SpC* wird eher nicht gesehen (etwas weniger von Ärzten), aber eben auch nicht eine besondere Nichtzuständigkeit. Hier drückt sich eine deutliche Zurückhaltung und Unsicherheit zur eigenen Positionierung aus. Eine „professionelle Neutralität“ ist auch bei US-amerikanischen Allgemeinmedizinern belegbar [7].
2. Für die Einschätzung einer besonderen *SpK* der eigenen Berufsgruppe sind Selbsterfahrung und proaktive Öffnung der beste Prädiktor, während die Nichtzuständigkeit besonders mit Umsetzungshemmnisses begründet wird, also eine eher abwehrende Haltung den *r/sp* Themen gegenüber. Hier spielt also die eigene (ablehnende) Grundhaltung eine bedeutende Rolle.
3. Für die Einschätzung einer besonderen *SpK* der eigenen Berufsgruppe ist Selbsterfahrung/proaktive Öffnung der beste Prädiktor, während die Nichtzuständigkeit besonders mit Umsetzungshemmnissen begründet wird, also eine eher abwehrende Haltung den *r/sp* Themen gegenüber widerspiegelt. Hier spielt also die eigene (ablehnende) Grundhaltung eine bedeutende Rolle. Die einzelnen *SpCK* sind unterschiedlich stark ausgeprägt und zeigen signifikante Unterschiede zwischen den in der Psychiatrie beschäftigen Berufsgruppen. Innerhalb der *SpCK* sind insbesondere Selbsterfahrung/Öffnung und Wahrnehmungs*-K* bei Gläubigen deutlich stärker ausgeprägt, während Hemmnisse eher bei den Wenig‑/Nichtgläubigen zu finden waren. Hinsichtlich der Gründe, die der Umsetzung von *SpC* im Wege stehen, unterscheiden sich die Berufsgruppen kaum. Die Pflegenden geben signifikant häufiger an, es sei „kein geeigneter Raum vorhanden“, die Ärzte, sie könnten „nicht alles lösen“.

Die meisten Autoren stimmen darin überein, dass *SpC* die Seelsorge nicht ersetzen, dass aber die dementsprechende Sensibilisierung und Kompetenzvertiefung der Gesundheitsberufe die Zusammenarbeit mit der Seelsorge erleichtern kann [22, 23]. Im Unterschied zum sehr restriktiven österreichischen Positionspapier [15] sieht das DGPPN-Positionspapier [29] einen größeren Spielraum vor, um die positiven, resilienzfördernden Möglichkeiten der Einbeziehung von *R/Sp* zu gewährleisten, ohne zu versäumen, auf mögliche Gefahren hinzuweisen. Ebenso wie das DGPPN-Positionspapier [29] versteht die Mehrheit der Literatur *Sp* als den im Vergleich zu *R* umfassenderen Begriff. Eine pflegewissenschaftliche Konzeptanalyse [5] subsummiert *Sp* als das wertebasierte Denken der Patienten, ihre Fähigkeit zur Interaktion mit anderen, ihre Suche nach Lebenssinn und Verbundenheit („connectedness“) mit anderen Menschen und mit dem transzendenten Heiligen. Psychiatrisches *SpC* könne tröstliche Effekte haben und das Coping der Patienten fördern. Eine aktuelle psychotherapeutische Studie [32] belegt die Zusammenhänge zwischen *R/Sp* einerseits und der Suche nach dem Lebenssinn und fordert, dass Psychotherapeuten ihren Patienten die Exploration von *R/Sp* ermöglichen, gleichzeitig jedoch ihre professionellen Grenzen wahren sollten.

*SpCK* im Sinne der in der vorliegenden Studie gemessenen Selbsteinschätzungen umfasst Wahrnehmung‑, Gesprächsführungs- und Dokumentations*-K* [13], klinisch gesprochen: die Fähigkeit zur Erhebung einer proaktiven *sp* Anamnese [29], die im deutschen Sprachraum noch zurückhaltend praktiziert wird [17]. Hingegen erklärten 93 % der von Curlin et al. [8] befragten US-amerikanischen Psychiater (vs. 53 % in anderen Facharztgruppen), es sei angemessen, *R/Sp* aktiv in der Anamnese zu explorieren. Eine polnische Studie [4] begründet die Zurückhaltung gegenüber der proaktiven *sp* Anamnese mit dem ethischen Argument des Patientenschutzes vor Grenzverletzungen und mit dem Mangel an diesbezüglichem Training.

Der häufig diskutierte *r/sp* „gap“ zwischen psychiatrischen Patienten und ihren Behandlern [6, 19] oder auch der weltanschaulichen „Kultur“ von Kliniken [11] wird dadurch vertieft, dass deutschsprachige Fachpersonen ihre *SpK* oft überschätzen und einschlägige Weiter- und Fortbildungsangebote noch immer dünn gesät sind [14]. Weder eine undifferenzierte Pathologisierung des *r/sp* Patientenerlebens noch die naive Idealisierung jedweder *Sp* sind in therapeutischer Hinsicht hilfreich [9]. Vielmehr setzt sich die Haltung durch, dass *SpK* zum kultursensiblen Umgehen mit Diversität gehört, das von den psychiatrischen Berufen zu fordern ist [16, 26]. Psychiatrisch Tätige brauchen *SpK* und psychopathologisches Urteilsvermögen, um *r*/*sp* Bedürfnisse einerseits, religiöse Wahnbildungen u. a. Krankheitssymptome andererseits zu unterscheiden und differenziert beantworten zu können [9]. Wenn z. B. das Verstummen *r/sp* Äußerungen zum Erfolgskriterium für die antipsychotische Medikation wird, verschließt sich ein unter Coping-Gesichtspunkten wichtiger Zugang zum Patienten [10]. Dazu bedarf es einer Verbesserung der Ausbildung [27, 28], der Weiterbildung [11, 28] und der Fortbildung [20, 25, 28, 31].

## Limitationen

Das Thema eines „personal Bias“ spielte in unserer Studie eine ebenso große Rolle wie in früheren Untersuchungen. Mit einer Selbstselektion ist schon bei der Entscheidung zur (Nicht‑)Teilnahme zu rechnen. Dieser Selektionseffekt geht allerdings auch mit einem Sensibilisierungseffekt einher: Das Bewusstwerden der eigenen *r/sp* Orientierung fördert das Einnehmen einer professionellen Haltung zu den *r/sp* Patientenbedürfnissen [10]. Es erleichtert dem Umgang mit sich wandelnden Definitionen von *R/Sp*: Während früher jegliche *Sp* im Kontext von *R* gesehen oder gar beide Terme gleichgesetzt wurden, wird nun zunehmend akzeptiert, dass es *sp* Bedürfnisse und Orientierungen auch außerhalb institutionalisierter *R* geben kann. Die damit gegebene begriffliche Unschärfe ist Vor- und Nachteil zugleich: Frühere Modelle wie die Gemeinschaftsspiritualität *r* gebundener Krankenschwestern oder die Gleichsetzung von wissenschaftlicher und atheistischer Haltung weichen weniger globalen und damit stärker personenbezogenen Zugangsweisen.

## Fazit für die Praxis

- *SpK* wird im Sinne der Patientenzentrierung gefordert (Matching zwischen *sp* Bedürfnissen psychisch Kranker und der diesbezüglichen Sensibilisierung der Behandler).
- Mithilfe des *SCCQ* können Maßnahmen der Aus‑, Fort- und Weiterbildung personen- und gruppenbezogen konzipiert und evaluiert werden.
- Verbesserung der *SpK* in der Psychiatrie kann durch Überarbeitung und Ergänzung von Lerninhalten und -zielen sowie durch Organisations- und Kulturentwicklung psychiatrisch-psychotherapeutischer Institutionen erreicht werden.
- Zentral ist die Förderung spezifischer diagnostischer Fähigkeiten, um auf *r/sp* Bedürfnisse und Krankheitssymptome differenziert einzugehen.
- Solange ein *r/sp* „personal bias“ des Behandlers unbewusst bleibt, kann es die Wahrnehmung verzerren. Wird es thematisiert und professionell gehandhabt, fördert es die Patientenorientierung.

## Abkürzungen

*K*: Kompetenz
*R*: Religion/Religiosität
*r*: Religiös
*SCCQ*: Spiritual Care Competency Questionnaire
*Sp*: Spiritualität
*sp*: Spirituell
*SpC*: Spiritual Care
*SpCK*: Spiritual-Care-Kompetenz
*SpK*: Spirituelle Kompetenz
